# 电磁导航支气管镜活检联合Massage染色定位在肺疾病诊疗中的应用

**DOI:** 10.3779/j.issn.1009-3419.2019.01.04

**Published:** 2019-01-20

**Authors:** 凯 钱, 涌耕 冯, 如文 王, 波 邓, 群友 谭

**Affiliations:** 400042 重庆，陆军军医大学大坪医院（野战外科研究所）；全军胸外科中心 Department of Thoracic Surgery, Institute of Surgery Research, Daping Hospital, Army Medical University, Chongqing 400042, China

**Keywords:** 电磁导航支气管镜, 活检, 肺周围型病变, 定位, Electromagnetic navigation bronchoscopy, Biopsy, Peripheral pulmonary lesion, Location

## Abstract

**背景与目的:**

电磁导航支气管镜（electromagnetic navigation bronchoscope, ENB）因无创、准确、实时等特点，是目前最新的微创诊疗技术。本研究探讨ENB活检联合一种新颖的Massage染色定位技术在肺周围型病变（peripheral pulmonary lesion, PPL）诊治中的应用。

**方法:**

回顾分析陆军军医大学大坪医院胸外科2017年8月-2018年1月施行ENB活检联合Massage染色定位的15例PPL患者的临床资料。其中，男性12例、女性3例，年龄（51.3±2.1）岁。

**结果:**

15例PPL直径6 mm-36 mm，平均14.0 mm。活检成功率66.7%。15例均成功行Massage染色定位。染色部位中心距离病灶中心（1.0±0.4）cm，染色扩散直径（2.8±0.6）cm。操作时间为（26.7±5.3）min，术中失血量（3.3±1.5）mL。操作过程中无气胸、大出血及气管损伤。

**结论:**

该技术损伤小、并发症率低、精度高、一站式完成诊断及定位，值得推广。

## Abstract (en)

**Background and objective:**

Electromagnetic navigation bronchoscopy (ENB) has become the latest minimally invasive diagnostic and therapeutic technique due to its characteristics, *e.g.*, non-invasion, accuracy, real-time positioning. In this study, we investigated the application of ENB biopsy combined with Massage staining in the diagnosis and treatment of peripheral pulmonary lesions (PPL).

**Methods:**

The clinical data of 15 PPL patients undergoing ENB biopsy plus Massage staining between August 2017 and January 2018 were retrospectively reviewed. Among them, there were 12 male and 3 female, and the mean age was (51.3±2.1) years old.

**Results:**

The diameter of PPLs ranged from 6 mm to 36 mm (mean: 14.0 mm). The successful biopsy rate was 66.7%. All patients successfully underwent Massage staining. The distance between the centers of staining and lesion was (1.0±0.4) cm, and the diameter of staining was (2.8±0.6) cm. The mean operation time was (26.7±5.3) min, and the mean blood loss during surgery was (3.3±1.5) mL. There was no pneumothorax, hemothorax and pulmonary vascular injury during the procedure.

**Conclusion:**

The ENB biopsy plus Massage staining technique caused very few complications, and provided high precision, which warrants further application.

随着胸部低剂量计算机断层扫描（computed tomography, CT）筛查的广泛应用，越来越多的肺周围型病变（peripheral pulmonary lesion, PPL）及磨玻璃病变（ground-glass node, GGN）被发现^[1]^。对于位置表浅、存在胸膜凹陷的PPL或实性结节，可以在术中视诊或采用手指触摸定位。但对于直径较小以及亚实性结节，手指及器械探查很难探及。术中依据CT影像判断肺部结节大致位置，病灶位置在肺萎陷前后常有偏差，导致术者无法准确判定其位置，无法精准切除，存在较大的医疗隐患。其他常用定位方法主要包括Hook-wire穿刺定位法、弹簧圈定位法以及CT引导下经胸壁穿刺美兰染色定位等，往往存在发生气胸、血胸及定位物脱落或移动等风险。

电磁导航支气管镜（electromagnetic navigation bronchoscope, ENB）是将电磁、3D影像和导航系统相结合，辅助准确到达肺外周并获取病变组织学诊断，是对传统纤维支气管镜、气管内超声以及气管内活检技术的补充^[2]^。术前利用ENB同步完成术前病理活检及病变定位，对于外科治疗肺部结节有重大的临床意义。

为进一步探索磁导航支气管镜技术在胸外科领域的应用，我们采用目前国内尚未开展的Massage染色定位法，对15例PPL患者实施了ENB活检及Massage染色定位，并同期切除病灶，效果良好。

## 1 材料与方法

### 1.1 一般资料

我科于2017年8月-2018年1月施行ENB活检联合Massage染色定位15例PPL患者（男性12例，女性3例）。年龄（51.3±2.1）岁。PPL直径6 mm-36 mm，平均14.0 mm。右上叶5例（33.3%），右中叶1例（6.6%），右下叶2例（13.3%），左肺上叶4例（26.6%），左下叶3例（20.0%）。15例中，实性结节6例（40.0%）、混杂性磨玻璃结节（mixed ground glass nodule, mGGN）5例（33.3%）、纯磨玻璃结节（pure ground glass nodule, pGGN）2例（13.3%）和空洞病灶2例（13.3%）。所有患者术前无病理学诊断。行ENB活检联合Massage染色定位，明确冰冻病理学结果后，同期手术治疗。根据患者症状、体征、术前检查评判，纳入标准为：①术前无明确病理诊断；②HRCT初筛检查发现肺部PPL，未经其他抗肿瘤治疗；③可耐受ENB操作；④排除手术禁忌证：肿瘤远处转移、出血倾向、凝血机制严重障碍、心肺功能不全、严重心律失常或高血压、重度肺动脉高压、呼吸道严重感染等。该研究经陆军军医大学大坪医院伦理委员会批准，所有入组患者术前获得告知，并签署知情同意书。

### 1.2 仪器设备

Super-D（AAS000161-02）电磁导航系统购自美国COVIDINE^TM^公司，包括1.9×1, 070 mm Edge^TM^定位引导丝、2.6×1, 050 mm引导丝扩展工作通道、1.8×1, 050 mm活检钳及1根1.8×1, 000 mm空心套管。电视胸腔镜手术（VATS）采用STORZ胸腔镜系统，机器人手术采用Da Vinci（达芬奇）机器人系统完成。

### 1.3 手术方法

全麻单腔气管插管，双肺通气。ENB传感器分别置于胸骨角及两侧腋前线第8肋间，经支气管镜吸痰及探查后，从纤维支气管镜工作孔道置入Edge定位引导丝及扩展工作通道，通过软件将匹配虚拟与实际支气管镜图像。由系统软件自动生成到达靶区的导航图（图 1A）。利用导航系统，操作者对引导丝位置进行校正并向前推进导丝，使引导丝到达病灶部位（图 1B）。随后退出引导丝，从导丝扩展通道置入活检工具，钳取出病变组织行冰冻病理学检查。再次置入定位导丝，根据导航系统指引到达病灶临近的胸膜处靶点（图 1C），置入与探头长度一致的1.8 mm直径套管，向套管内注入亚甲蓝（图 1D），注入剂量为0.8 mL/cm病灶直径。再次置定位引导丝，再次确认已到达胸膜设定部位后反复多次抽送导丝，完成Massage染色定位（图 1E、图 1F）。根据图 2策略，决定下一步手术方式。

**1:**
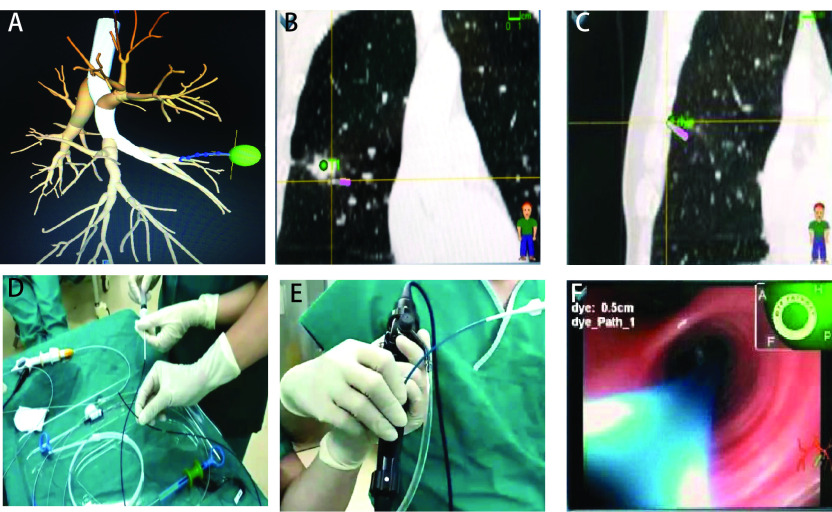
电磁导航支气管镜活检联合Massage染色定位操作步骤。A：制定术前计划，由计算机软件自动生成到达靶区的导航图；B：定位导丝及扩展工作通道到达病灶部位；C：定位导丝及扩展工作通道到达脏层胸膜；D：将美兰溶液预充在1.8 mm的导管中；E：将预充美兰溶液的导管置入扩展工作通道进行Massage染色，红色箭头方向为Massage染色过程中导管搓揉的方向；F：定位导丝到达脏层胸膜。 Electromagnetic navigation bronchoscopy biopsy combined with massage staining positioning steps. A: Develop a pre-operative plan to automatically generate a navigation map to the target area by computer software; B: Positioning guide wire and extended working channel reached the lesion; C: Positioning guide wire and extended working channel reached the visceral pleura; D: The methylthionine chloride was filled in the 1.8 mm catheter; E: The catheter with methylthionine chloride was placed into the extended working channel, and the direction of catheter rubbing during Massage staining was shown by the red arrow; F: Positioning guide wire reached the visceral pleura.

**2:**
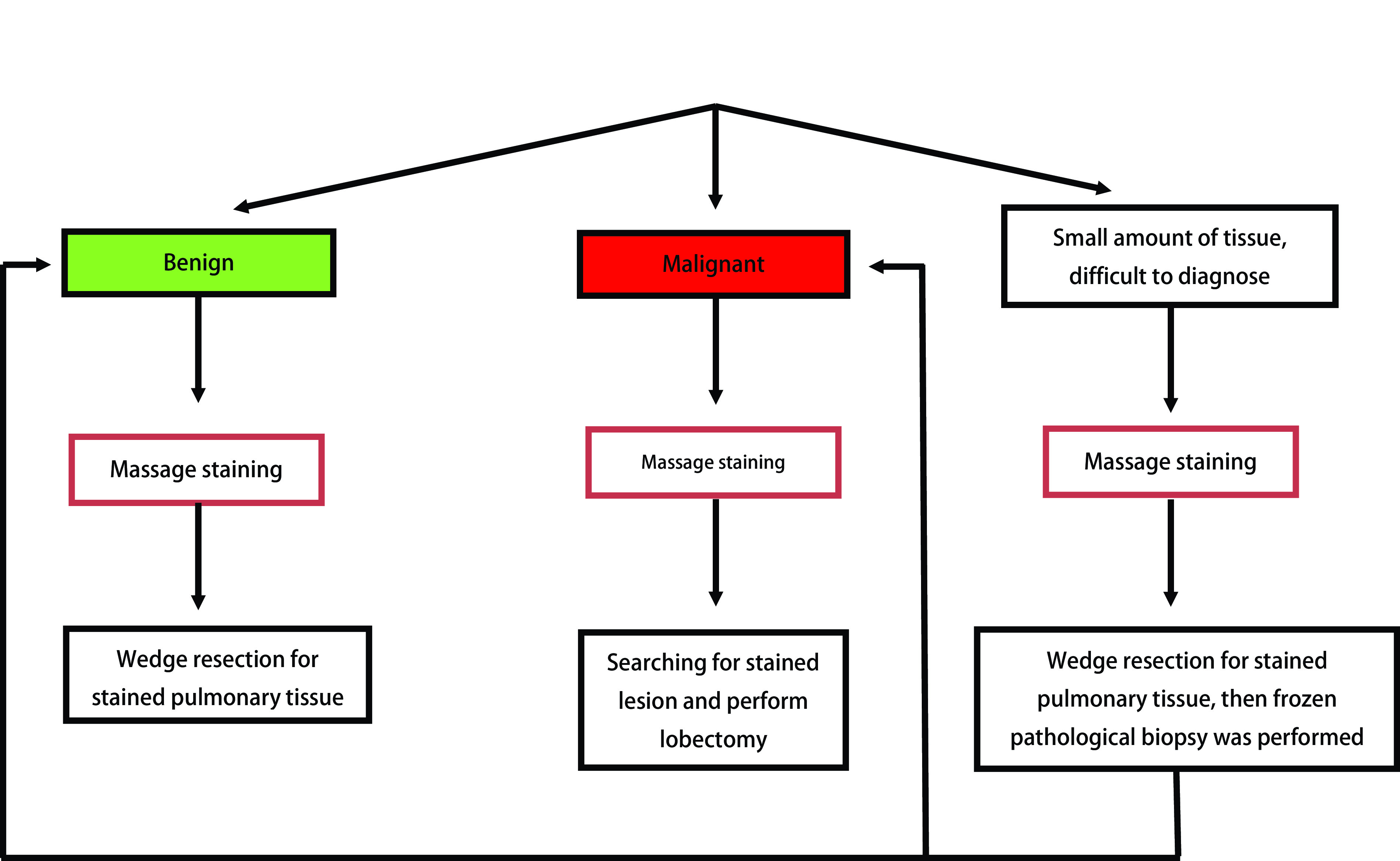
ENB活检联合Massage染色定位后手术方式选择策略 Strategy of surgical treatment following ENB biopsy plus Massage staining. ENB: electromagnetic navigation bronchoscope.

## 2 结果

10例患者（恶性肿瘤6例，良性病变4例）术中冰冻活检诊断与术后病理诊断一致，总诊断率为66.7%。其余5例中，2例因支气管炎症闭锁，未能到达病变位置；3例虽到达病变位置，由于获取组织过少，未能获得冰冻诊断。

定位染色过程中，13例患者顺利到达脏层胸膜行Massage染色定位（图 3A），2例因炎症导致终末支气管阻塞，引导丝无法进入，在距脏层胸膜0.8 cm及0.9 cm行Massage染色（图 3B）。所有患者定位点均与实际病灶的位置相符，染色部位中心距离病灶中心平均距离为（1.0±0.4）cm，染色扩散直径为（2.8±0.6）cm，胸腔镜下观察染色部位清晰易识别。根据术中病理结果，5例诊断为炎性假瘤，故行胸腔镜楔形切除术，1例患者术前经规范抗感染治疗无效，ENB活检诊断为慢性病变，结合病史影像学考虑机化性肺炎，行VATS肺叶切除术。9例术中诊断为非小细胞肺癌，其中8例行VATS肺叶切除术及淋巴结清扫术，1例行机器人肺叶切除术。在ENB活检及Massage染色术中无气胸、大出血及气管损伤等。平均操作时间为（26.7±5.3）min，术中平均失血量为（3.3±1.5）mL。

**3:**
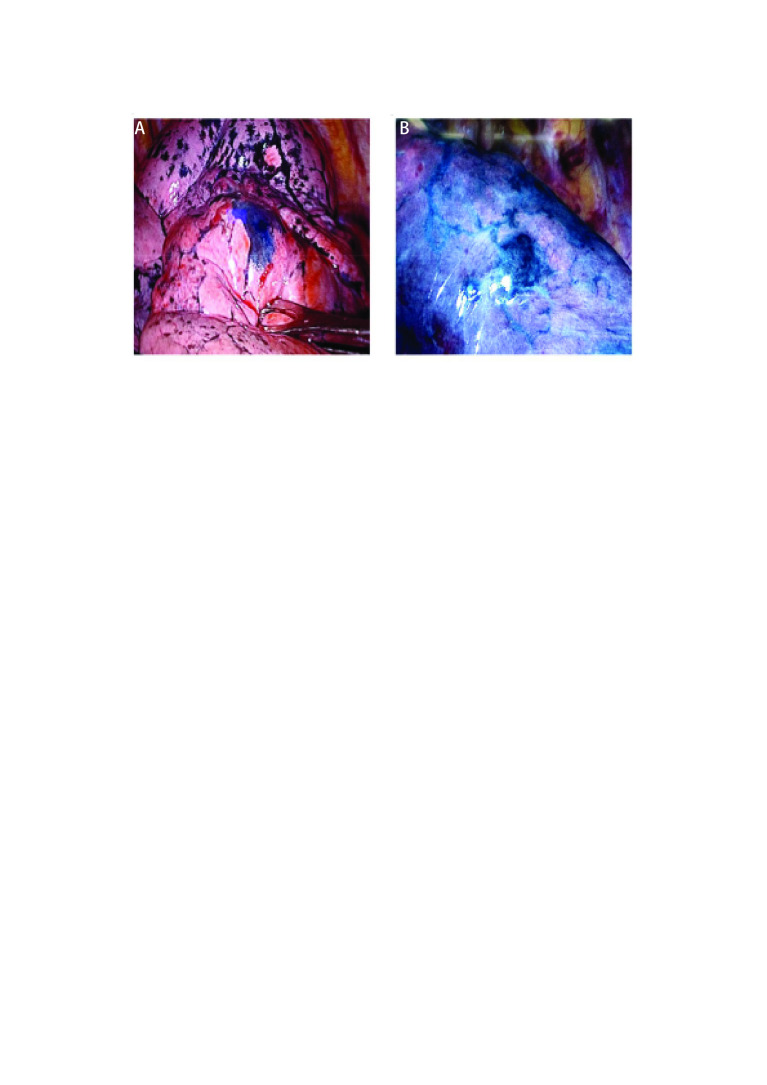
Massage染色效果。A：套管到达脏层胸膜Massage染色效果；B：套管距离脏层胸膜约0.8 cm时Massage染色效果。 Effect of massage staining. A: The effect of Massage staining where the catheter reaches the visceral pleura; B: The effect of Massage staining where the catheter from the visceral pleura is about 0.8 cm.

## 3 讨论

随着肺癌筛查逐渐被重视，越来越多的早期肺部结节被发现。明确病理诊断对选择手术方式至关重要。PET/CT其诊断敏感性和特异性分别仅为88%、77%^[3]^。CT引导经皮肺穿刺活检准确率并不尽如人意，此外穿刺有可能导致气胸及肿瘤转移^[4]^。超声支气管镜引导下的经支气管针吸活检（transbronchial needle aspiration biopsy guided by ultrasound bronchoscop, EBUS-TBNA）的检出率与肿瘤大小及部位密切相关：病灶 > 2 cm，检出率约为63%；病灶 < 2 cm，降为34%^[5]^。

ENB原理是系统对照CT虚拟的3D支气管镜图像，形成“路线”，在磁导航的引导下，操作者将直径约2 mm工作通道和导丝送至病灶部位，可到达脏层胸膜水平的病灶。置入相应的活检器械如活检钳、活检针或细胞刷对病灶进行活检^[6]^。多中心前瞻性研究显示ENB活检的成功率可高达91.8%^[2]^。对于常规支气管镜无法到达或无法耐受肺穿刺的患者，ENB更具有优势。

目前较为常用的术中病灶定位方法包括：CT引导下经皮肤穿刺钩线定位技术（Hookwire）、注射染料、对比剂或放射性核素、植入弹簧圈以及术中使用超声或可移动CT装置等。Kleedehn^[7]^比较了美兰穿刺染色和Hookwire穿刺定位，并发症发生率分别高达54%和46%，13% Hookwire定位点发生移位。我们采用Massage染色定位法，利用ENB引导套管准确地到达脏层胸膜内侧，在ENB实时监测下，将含有亚甲蓝的导管与胸膜间摩擦，使脏层胸膜染色。由于脏层胸膜完整性未遭到破坏，可以有效限制染色范围，提高了手术切除的精确性，并且降低了气胸及血胸的发生率。

常用ENB定位染色剂有亚甲蓝、靛卡红和生物蛋白胶等。靛卡红在染色定位后3天仍可辨认，而亚甲蓝在染色数小时后消散。因此，对于染色定位后可立即手术的患者，亚甲蓝和靛卡红染色均可，而染色后不能立即手术的患者应首选靛卡红作为标记物^[8]^。有报道^[9]^将亚甲蓝和生物蛋白胶混合，生物蛋白胶在气道内可减缓亚甲蓝的扩散速度，同时形成可触及的蓝染区域，但相对繁琐且受材料限制。我们尝试采用亚甲蓝进行Massage染色，对脏层胸膜进行局部点状摩擦，由于染色剂剂量较少，摩擦后染色剂不会产生大的弥散面积，能很好地分辨病变区域，帮助我们术中快速便捷寻找病灶。

**1:** 患者一般情况 Demographical-clinical characteristics of patients in this study

Variables	Number of cases
Gender (male:female)	12:3
Age (yr)	51.3±2.1
Massage staining	15
Diameter of lesions (mm)	14.0±2.5
ENB biopsy	15
Mode of operation	
Pulmonary wedge resection	5
VAST-assisted pulmonary lobectomy	9
Robotic-assisted pulmonary lobectomy	1
Conventional pulmonary lobectomy	0

本研究我们总结出以下经验：①Massage染色定位的关键在于尽可能地使导管尖段到达脏层胸膜，对于导管未到达脏层胸膜采用Massage法染色后会出现染色剂扩散，但患者采用Massage染色后仍能准确辨认出病变部位。②应尽可能减少染色剂的剂量，Massage染色定位法通过导管与胸膜的点状摩擦，可有效地标记病变位置并减少染色剂的扩散。

**2:** 病灶特点与Massage染色效果 Characteristics and the dyeing effects of peripheral pulmonary lesions in this study

Characteristics	Total	Dye(+)	Dye(-)
Size of nodule (mm)			
≤10	4	3	1
11-15	6	6	0
16-0	4	4	0
≥21	1	0	1
Character of nodule on CT finding			
Solid nodule	6	5	1
mGGN	5	5	0
pGGN	2	3	1
Cavitary nodule	2	2	0
Dye (+):Good dyeing effect and accurate lesion location；Dye (-): After dyeing, the dyeing agent is dispersed, but the dyeing effect is not satisfactory.			

综上所述，ENB下Massage染色定位技术对肺组织损伤小，并发症发生率低，精度高，可一次性完成诊断及定位，是一项安全、高效的操作，能提高微创外科PPL精确切除的成功率。但本研究样本量偏小，需不断总结经验，并扩大样本量，进一步验证及完善Massage染色定位技术。
